# Biofluid Biomarkers in Frontotemporal Lobar Degeneration (FTLD)

**DOI:** 10.1002/alz70856_100374

**Published:** 2025-12-25

**Authors:** Carmela Tartaglia

**Affiliations:** ^1^ Krembil Brain Institute, University Health Network, Toronto, ON, Canada; Tanz Centre for Research in Neurodegenerative Disease, University of Toronto, Toronto, ON, Canada; Toronto Dementia Research Alliance, Toronto, ON, Canada

## Abstract

Frontotemporal lobar degeneration (FTLD) is the underlying pathological substrate of frontotemporal dementia (FTD) and related syndromes. These syndromes feature diverse clinical presentations—behavioral, cognitive, and motor deficits—and significant pathological heterogeneity, with most cases linked to tau or TAR DNA‐binding protein 43 (TDP‐43), and 5% related to fused in sarcoma (FUS). While most cases of FTLD are sporadic, around 10–20% of patients harbor an autosomal dominant mutation, typically in *MAPT* (tau), *GRN* (progranulin), or *C9orf72*. Detection of FTLD often occurs at advanced disease stages, limiting the opportunity for timely intervention. Early detection is critical for implementing disease‐modifying therapies, yet there remains a pressing need for diagnostic biomarkers capable of identifying the specific underlying pathology, particularly tau and TDP‐43, in the early stages of the disease.

Fluid biomarkers have played a transformative role in advancing therapeutic strategies for Alzheimer's disease (AD), providing a roadmap for biomarker‐driven research in FTLD. This session will explore the state‐of‐the‐art developments in biomarkers for FTLD. Beyond CSF and plasma, innovative approaches such as skin biopsies are being investigated for detecting pathological aggregates of tau, TDP‐43, or related molecular changes. These biomarkers hold potential for both detecting FTLD and differentiating between its major pathological subtypes: FTLD‐tau and FTLD‐TDP‐43. Biomarkers such as neurofilament light chain (NfL) are robust but non‐specific markers of neurodegeneration and so lacks specificity for FTLD or its pathological subtypes. Proteomic and metabolomic approaches have recently enabled the discovery of novel candidate biomarkers that could aid in more specific differentiation. Emerging data suggest that markers of neuroinflammation and synaptic dysfunction may offer greater specificity for FTLD subtypes. Biomarkers in FTLD are not only pivotal for diagnosis but also for tracking disease progression and monitoring response to emerging therapies. A comprehensive biomarker panel, incorporating markers of neurodegeneration, neuroinflammation, and specific pathology, may eventually enable personalized treatment approaches as well as evaluate the role of co‐pathology.

